# The effects of all trans retinoic acid, vitamin D3 and their combination on plasma levels of miRNA-125a-5p, miRNA-34a, and miRNA-126 in an experimental model of diabetes

**DOI:** 10.22038/AJP.2021.18598

**Published:** 2022

**Authors:** Mohammad Sharifzadeh, Aghil Esmaeili-Bandboni, Mohammad Reza Emami, Fatemeh Naeini, Meysam Zarezadeh, Mohammad Hassan Javanbakht

**Affiliations:** 1 *Department of Clinical Nutrition, School of Nutritional Science and Dietetics, Tehran University of Medical Sciences (TUMS), Tehran, Iran*; 2 *Department of Medical Genetics, School of Medicine, Guilan University of Medical Sciences, Rasht, Iran*; 3 *Cellular and Molecular Research Center, School of Medicine, Guilan University of Medical Sciences, Rasht, Iran*; 4 *Student Research Committee, Tabriz University of Medical Sciences, Tabriz, Iran *; 5 *Nutrition Research Center, Department of Clinical Nutrition, School of Nutrition and Food Sciences, Tabriz University of Medical Sciences, Tabriz, Iran*; 6 *Department of Cellular and Molecular Nutrition, School of Nutritional Sciences and Dietetics, Tehran University of Medical Sciences (TUMS), Tehran, Iran*

**Keywords:** Plasma miRNAs, miR-126, Type 2 diabetes, Cardiovascular disease

## Abstract

**Objective:**

The purpose of this study was to evaluate the effects of ATRA (all trans retinoic acid), vitamin D3, and their combination on circulating levels of miR (MicroRNA) -125a-5p, miR-126, and miR-34ain diabetic rats.

**Materials and Methods:**

Total miRNA was extracted from plasma samples. miRNA expression profiles of 30 rats in five groups were analyzed after 4-week intervention. The expression levels of miRNAs were measured using qRT-PCR.

**Results:**

We analyzed the expression of miR-126, miR-125a-5p, and miR-34a in serum among all five groups (p=0.268). The levels of miRNA-126 (p=0.004) and miR-125a-5p (p=0.014) showed a significant difference among our experimental groups. The circulating levels of miR-126 decreased in DC (Diabetic control) group compared to the HC (Healthy control) group (p=0.009). In addition, vitamin D3+ATRA supplementation increased miR-126 expression (p=0.014). Moreover, the levels of miR-125a-5p decreased in the DC group compared to the HC group (p=0.019).

**Conclusion:**

The expression of miR-126 and miR-125a-5p decreased in diabetic rats. Also, vitamin D3+ATRA can be considered a new therapeutic agent that can elevate miR-126 expression and prevent diabetes-related cardiovascular complications.

## Introduction

Diabetes mellitus is a metabolic disorder characterized by chronic hyperglycemia and disturbances in macronutrient metabolism especially carbohydrates due to defects in insulin secretion, insulin action, or both (Gonzalez et al., 2020 ▶). Globally, the prevalence of diabetes is increasing and the number of diabetic patients is 422 million in 2020 (Lovic et al., 2020 ▶). The risk of cardiovascular disease and infarction was reported to be similar between diabetic patients who did not have a history of myocardial infarction (MI) and non-diabetic patients with a prior MI (Haffner et al., 1998 ▶). Under oxidative stress status, LDL (low density lipoprotein)-cholesterol converts to its oxidized form (ox-LDL) and plays a pivotal role in the progression of atherosclerosis. In diabetic patients, a remarkable positive correlation was observed between serum ox-LDL levels and the length of diabetes, while no alteration in LDL-cholesterol concentrations was found (Nakhjavani et al., 2010 ▶). Atherosclerotic plaque formation is accelerated by ox-LDL and it may contribute to the expansion of microvascular complications in diabetes (Wegner et al., 2012 ▶). Atherosclerosis lesions (atheroma), asymmetric thickenings of the internal layer of the artery (intima) in the focal area, consist of cells, lipids, connective tissue elements, and debris. Atherosclerosis can develop in response to endothelial cell (EC) injury caused by many different stimuli including diabetes, dyslipidemia, and hypertension (Rudijanto, 2007 ▶). Moreover, it should be noticed that atherosclerosis is one of the main leading causes of death in cardiovascular diseases (Stöger et al., 2012 ▶). Thus, diabetes can increase the risk of cardiovascular abnormalities and cardiac-related mortality through induction of an increase in serum ox-LDL concentrations and an elevation in the formation of atherosclerotic plaques.

MicroRNAs (miRNAs), short noncoding RNAs, consist of about 18 to 26 nucleotides that posttranscriptionally suppress gene expression via degradation or translational inhibition of their target mRNAs. There is strong evidence suggesting that miRNAs are involved in nearly all pathological and physiological processes. It was revealed that the expression profiles of microRNAs changed in cancer and diabetes (Qin et al., 2011 ▶). The plasma concentrations of miRNAs vary in different conditions including diabetes. Because of their measurable nature, stability, and non-sensitivity to RNAse, miRNAs can be used as a marker for identification of various diseases (Chen et al., 2008 ▶).

The miRNA-34a level increases in human atherosclerosis plaque and in the serum of diabetic patients (Kong et al., 2011 ▶). Previous studies exhibited that the level of miRNA-34a was significantly higher in the serum of diabetic patients compared to pre-diabetic and non-diabetic patients (Kong et al., 2011 ▶; Raitoharju et al., 2011 ▶). The plasma levels of some cytokines including interleukin (IL)-1β, interferon (IFN)-γ, and tumor necrosis factor α (TNFα) increase significantly in patients with diabetes (King, 2008 ▶). IL-1β and TNF-α up-regulate mir-34a expression (Roggli et al., 2010 ▶). Furthermore, an *in vitro* model elucidated that palmitate, the most common saturated fatty acid leads to a systemic inflammatory response, and could suppress b-cell lymphoma 2 (Bcl 2) gene expression and destroy pancreatic beta cells by up-regulating miR-34a (Gurzov andEizirik, 2011 ▶).

miR-125a-5p contributed to a considerable decrease in the secretion of inflammatory cytokines and inhibiting lipid uptake by macrophages which are stimulated by ox-LDL (Chen et al., 2009 ▶). Endothelin-1 (ET-1) increases the uptake of ox-LDL by stimulating macrophages and because the ET system is involved in vascular dysfunction and progression of cardiovascular diseases, it can be considered a destructive factor (Böhm andPernow, 2007 ▶). An increase in miR125a-5p production can decrease the ET-1 levels and therefore, miRNA-125a-5p plays a protective role against the mentioned complications (Zhang andWu, 2013 ▶).

Moreover, miR-126 enhances the proangiogenic actions of VEGF (vascular endothelial growth factor) and FGF (fibroblast growth factor) and promotes blood vessel genesis by down-regulation of spred-1, an intracellular inhibitor of angiogenic signaling (Wang et al., 2008 ▶). High plasma glucose concentrations impaired proangiogenic properties of miR-126 in diabetic patients by reducing miR-126 content of endothelial and decreasing miR-126 levels of CD34+ PBMCs (peripheral blood mononuclear cells) (Meng et al., 2012 ▶; Zampetaki et al., 2010 ▶; Mocharla et al., 2013 ▶). 

A growing body of evidence disclosed that vitamin D and ATRA (all trans retinoic acid) decrease plasma concentrations of inflammatory cytokines and vitamin D attenuates ox-LDL uptake by macrophages (Nozaki et al., 2006 ▶). Furthermore, higher levels of ox-LDL were observed in diabetic patients with vitamin D deficiency (Gradinaru et al., 2012 ▶). Vitamin A which is a micronutrient plays a crucial role in maintaining vision, development, and the immune system and mucus integrity (Pino‐Lagos, Guo, and Noelle, 2010 ▶). This vitamin exists in animal products and it is produced from various precursors that come from plants and vegetables like carrot (Meléndez‐Martínez, 2019 ▶). It is available in different forms of retinal, retinol, and retinoic acid (RA), among which RA shows the highest biological activity. RA has two different forms named 9-cis-RA and ATRA, both are able to influence gene expression (Ross et al., 2000 ▶).

Vit A and D, either alone or in combination, may be useful to prevent and control the risk factors of diabetic atherosclerosis. Due to the high prevalence of diabetes and its-related disorders including atherosclerosis worldwide and lack of adequate studies investigating the effects of vitamin A and D and their combination on plasma level miRNAs which are mentioned as factors involved in diabetes-related complications, the present study aimed to determine the effect of vitamin A and D, and their combination on plasma levels of miR125a-5p, miR-34a, and miR-126in Sprague-Dawley rats. 

## Materials and Methods

**Diabetic rat model**

In this study, young adult Sprague–Dawley rats (180-260 g) were purchased from Pharmacy College of Tehran University of Medical Sciences, kept for 48 hr without intervention to adapt to the environment and fed with standard rat chow diet with a 12:12-hr light-dark cycle. The current study was performed according to the animal care protocol, approved by the animal care and research committee of Tehran University of Medical Sciences (ethical number: 9411468009). Diabetes was induced by a single injection of 60 mg STZ (streptozotocin)/kg body weight intraperitoneally (Sigma, St. Louis, MO) in 1 mM phosphate buffer, blood glucose levels were measured immediately before and 2 days after STZ injection. Animals with blood glucose levels>200 mg/dl were deemed diabetic. Rats became hyperglycemic 2 days after STZ injection and maintained hyperglycemic before being killed for blood collecting after the onset of diabetes.

**Intervention**

Two control groups were used: group 1 (the healthy control group that received normal saline 0.5 ml), and group 2 (the diabetic control group that received vehicle flaxen oil). In ATRA-treated group (group 3), we injected 12.5 mg/kg ATRA (manufactured by Sigma Corporation of America SIGMA R2625) dissolved in 0.5 ml of flaxen oil intraperitoneally (1 injection every other day). Group 4 received intraperitoneal doses of 5000 IU/kg of vitamin D3 in 0.5 ml of flaxen oil (1 injection per week). Group 5 received intermittent administration of a combined dose of 12.5 mg/kg of ATRA (1 injection every other day) and 5000 IU/kg of vitamin D3 that were dissolved in 0.5 ml of flaxen oil on a weekly basis.

**Experimental procedure**

The rats were divided into five groups as follows. Five animals were in group I, IV, and V and six animals were in group II, and III. Group I: Healthy control rats; Group II: STZ-induced diabetic control rats; Group III: Diabetic rats given ATRA (12.5 mg/kg) in 0.5 ml flaxen oil solution every other day using an intraperitoneal injection for 30 days; Group IV: Diabetic rats given vitamin D3 (5000 IU/kg) in 0.5 ml flaxen oil solution every week using an intraperitoneal injection for 4 weeks and Group V: Diabetic rats given vitamin D3 (5000 IU/kg/week) + ATRA (12.5 mg/kg/ every other day) in 0.5 ml flaxen oil solution using an intraperitoneal injection for 30 days.

**Blood samples**

At the end of the fourth week, 12 hr prior to the killing of the animals, their food was taken for fasting. After weighing and measuring blood glucose in rats, each of them was first anesthetized using ketamine and xylazine and then sacrificed peripheral blood specimen (6 ml) was drawn from each rat. EDTA (ethylenediamine tetraacetic acid) was used as an anticoagulant. Plasma was separated by centrifugation at 1200×g for 10 min at 4°C, and stored in 1.5 ml RNAse-free tubes at –80°C.

**Quantitative real** **‐** **time polymerase chain reaction (qRT** **‐** **PCR) detection of miRNAs expression in plasma**

Total RNA was extracted using Hybrid-R miRNA isolation Kit (GeneAll, South Korea) according to the manufacturer's instruction. Concentration and purity of the extracted miRNA were evaluated by a Nano Drop and samples were stored at -80°C until analysis. cDNAs were synthesized from 5uL of total miRNA using PrimeScript® 1st strand cDNA Synthesis Kit (TaKaRa Bio, Siga, Japan) according to the manufacturer’s protocol. The expression of miRNAs in samples and controls was detected by using miScript SYBRGreen PCR kit (TaKaRa Bio, Shiga, Japan). The reaction was performed in a real-time quantitative PCR machine (Applied Bio system step one plus). PCR was carried out using the first chain of cDNA as the pattern. The reaction system contained 2 L of cDNA, 10 L of SYBR Green I Master mix, 10 µl of miRNA-specific primers, 1 µl of upstream universal primer, 6 µl H_2_O, and 0.5 Rox. A mean of triplicate findings was used as the result for every sample. miR-16 was used as an endogenous reference for normalization. Relative changes of gene expression were calculated by 2-ΔΔ Ct which is defined as follows: △△Ct= (CtmiRNAs-CtmiR16) of the treatment group- (CtmiRNAs-CtmiR16) mean of the control group.

**Glucose, insulin and vitamin D assay**

The glucose levels were measured by glucose oxidase method, using glucose kit (biochemistry, Tehran). Also, insulin levels were measured using ELISA kit (DiaMetra, Perugia Italy). In addition, 25-hydroxyvitamin D levels were measured by radioimmunoassay method, using a kit (Belgium BioSource).

**Statistical analysis**

Graph Pad Prism V4.03 and SPSS16.0 were used for statistical analysis. We used the Kruskal-Wallis test for comparison among multiple groups. A p<0.05 was considered statistically significant.

## Results

To explore whether miRNAs are associated with diabetes or not, four of them including miR-125a-5p, miR-126, and miR-34awhich are assumed to relate to diabetes were detected in the plasma of experimental rats using qRT–PCR. 

We compared the expression of plasma miRNAs in five independent samples by nonparametric Kruskal Wallis test. The findings revealed the differences in expression of miR-126 (p=.004), miR-125a-5p (p=.014), and miR-34a (p=.268) between all five groups (ATRA, ATRA+vitamin D3, vitamin D3, healthy control, and diabetic control).

**Figure 1:**
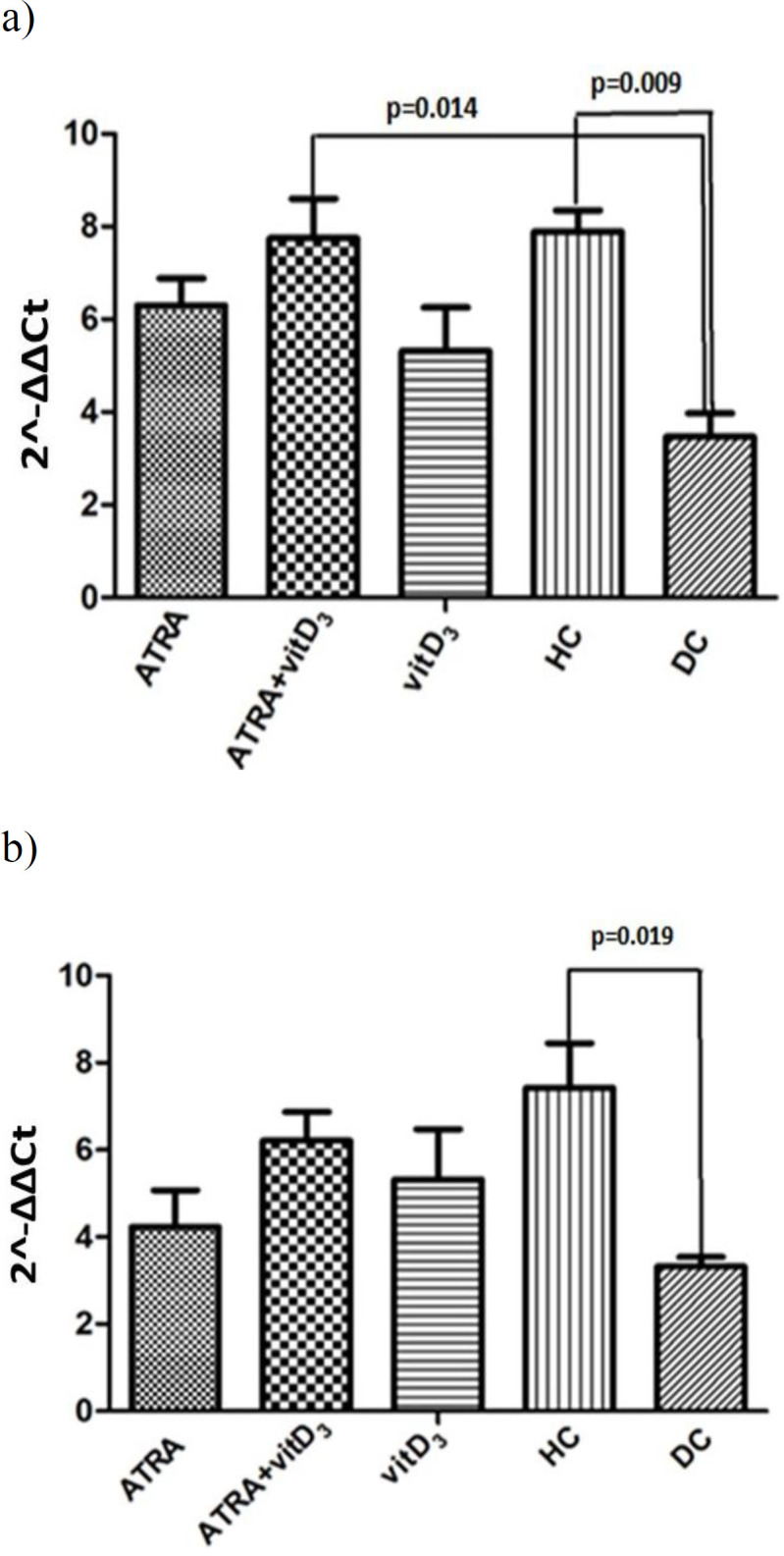
Circulating levels of miR-34a, miR-126, and miR-125a-5p in serum samples of HC (healthy control), DC (diabetic control), vitamin D3, ATRA (all Trans retinoic acid) AND ATRA + vitamin D3 groups. qRT-PCR was performed for the extraction of miRNAs from plasma samples of five groups

Based on the obtained results, miRNA-126 and miR-125a-5p levels showed significant differences in our experimental groups. The levels of miR-126 decreased in DC (Diabetic control) group compared to the HC (Healthy control) group (p=0.009) and its expression increased by vitamin D3+ATRA supplementation (p=0.014) (Figure 1a). Also, mir-125a-5p decreased in the diabetic group compared to the HC group (p=0.019) (Figure 1b). 

The mean plasma levels of 25-hydroxyvitamin D3, fasting insulin, and fasting blood glucose are reported in Table 1. After induction of diabetes and before the intervention, the mean fasting blood glucose levels were significantly higher in all diabetic groups compared to the HC group (p˂0.05). Whereas, no significant difference was found in plasma levels of glucose among the diabetic groups. Similarly, at the end of the study (day 28), mean fasting blood glucose levels were significantly higher in all diabetic groups compared to the HC group (p˂0.05). However, there was no significant difference in plasma glucose levels among diabetic groups. After the intervention, fasting insulin levels were higher in all intervention groups compared to the DC group; however, the differences were not statistically significant. In case of 25-hydroxyvitamin D3, the mean plasma levels in ATRA, HC, and DC groups were significantly lower than the groups that received vitamin D3 or the combination of ATRA and vitamin D3 (Table 1).

**Table 1:** Plasma glucose, insulin, and vitamin D concentrations of rats at baseline and after intervention

variable	Groups							
	ATRA	ATRA/Vit.D		Vit.D	DC	HC	p value	
Vitamin D (ng/ml)	45.96±3.80^a^		56.47±6.61	57.43±2.41	41.29±10.64^a^	40.23±12.^a^		0.004
Fasting Insulin (µIU/ml)	5.41±0.29^b^		5.84±0.76^b^	6.16 ±0.71^b^	4.77±0.59	5.67±0.56^b^		0.017
Fasting plasma glucose (mg/dl) before	330±109.3^c^		272 ±84^c^	288.8±79.5^c^	300.3 ±868^1c^	96.2±14.7		0.001
Fasting plasma glucose (mg/dl) after	264.1±119.5^d^		277.5±188.3^d^	343.4±124.5^d^	379.2± 130.2^d^	92.4 ± 14.3		0.016

a. The mean values±SD (standard deviation) are statistically significantly higher than the diabetic control group (p˂0.05). b. The mean values±SD (standard deviation) are statistically significantly higher in comparison with the diabetic control group (p˂0.05). c. The mean values±SD (standard deviation) are statistically significantly higher than the diabetic control group (p˂0.05).

## Discussion

In recent decades, miRNAs were one of the most interesting issues in nutrition and medical science research. miRNAs have regulatory functions in the physiological and biological processes by post-transcriptional regulation of gene expression and RNA silencing (Macfarlane andMurphy, 2010 ▶). Presently, hundreds of human miRNAs are defined in miRBase (Alles et al., 2019 ▶). The present study aimed to investigate the effects of ATRA, vitamin D3, and their combination on circulating levels of miR-125a-5p, miR-126, and miR-34aas new biomarkers or therapeutic targets for diabetes instead of existing traditional biochemical markers including HbA1c and fasting plasma glucose concentrations. In order to select proper miRNAs for this research project, we reviewed the relevant articles and finally selected the aforementioned miRNAs which have been suggested to be involved in the pathophysiology of diabetes (Tang, Tang, and Ozcan, 2008 ▶). Major findings of the current study indicated significant differences in miR-126 and miR-125a-5p levels in all groups and vitamin D3+ATRA supplementation increased miR-126 expression, while no significant effect was observed when vitamin D3 or ATRA was administered separately. 

Lin et al. (Lin et al., 2014 ▶) reported that plasma miR-34a concentration was significantly elevated in diabetic patients compared to pre-diabetic and non-diabetic patients. Expression of miR-34a rose after exposure to palmitate in the pancreatic islets and b-cell line which led to suppression of Bcl-2 expression and induction of B cell apoptosis (Lin et al., 2014 ▶). However, our results demonstrated that serum miR-34a levels did not change significantly in DC group compared to the HC group. It should be noted that different methods and primers which are used to determine the plasma levels of miR-34a may explain the discrepancy between findings.

High glucose concentration in diabetic patients reduced the miR-126 content of endothelial and CD34+ PBMCs which increased the risk of diabetes-related cardiovascular diseases through impairing miR-126 proangiogenic properties (Meng et al., 2012 ▶). In consistence with our findings, serum miR-126 levels considerably decreased in subjects with impaired glucose tolerance (IGT) and impaired fasting glucose (IFG) and in patients with type 2 diabetes. However, miR-126 expression increased significantly after treatment with antidiabetic drugs and life style change for six months. Thus, miR-126 may be serve as a potential biomarker for the early identification of individuals who are susceptible to diabetes (Liu et al., 2014 ▶; Zhang et al., 2013 ▶). The biological activities of retinoic acid are mediated through the retinoic acid receptor (RAR) along with the retinoid X receptor (RXR) that is commonly composed of RXR/RAR heterodimers. Also, vitamin D signaling is transduced by RXR and vitamin D receptor (VDR) heterodimers. Therefore, ATRA and 1, 25-dihydroxyvitamin D3 have synergistic effects due to the use of RXR as the common receptor (Chambon, 1996 ▶; Collins, 2002 ▶; Zechel, 2005 ▶). As confirmed by our results, a significant synergistic increase in the expression of miR-126 was found when ATRA and vitamin D3 were supplemented in combination and not separately. Accordant to the mentioned result, Jorde et al (Jorde et al., 2012 ▶) reported that vitamin D3 administration (40000 IU per week) for 12 months did not affect the miRNA plasma levels significantly.

Previous studies demonstrated that miR-125a-5p remarkably decreased in patients with breast cancer and non-small-cell lung cancer (NSCLC), therefore, it might be used as a useful noninvasive biomarker for the clinical diagnosis and treatment (Hsieh et al., 2015 ▶; Wang et al., 2015 ▶). Moreover, a growing body of evidence exhibited that reduction in miRNA-125a-5p expression level attenuated the phenomenon of atherosclerosis, one of the most important diabetes-related cardiovascular abnormalities, through inhibiting the secretion of inflammatory cytokines and lipid uptake by macrophages that were stimulated by ox-LDL (Churov et al., 2019 ▶; Chen et al., 2009 ▶). A recent study performed by Xu et al (Xu et al., 2018 ▶) concluded that miRNA-125a-5p, a regulator of glycolipid metabolism, should be considered a therapeutic target for diabetes due to the inhibition of hepatic lipogenesis and gluconeogenesis and elevation of glycogen synthesis through targeting STAT3 in diabetic rats and mice.

The mechanism of action of vitamin D3 on miRNAs is explained by direct VDR element-mediated modulation. Canonical VDR-mediated modulation of miRNAs through VDR elements has been discovered for different kinds of miRNAs (Giangreco andNonn, 2013 ▶). Also, retinoic acid affects miRNAs by several mechanisms including regulation of Bcl-2 and RAS (Rat sarcoma) proteins family expression and modulation of nuclear factor-kappa B (NF-kB) pathway (Garzon et al., 2007 ▶).

There are some limitations in our research. First, the short duration of intervention (4 weeks) may lead to therapeutic effects decreased. Second, the small sample size of our study may lead to bias in the analysis. Third, due to the experimental nature of the present study, the findings may be imprecise to generalize to humans.

In summary, the expression of two miRNAs miR-126 and miR-125a-5p decreased significantly in diabetic rats. Therefore, these miRNAs may be considered biomarkers for early identification of diabetes. Also, our findings suggested that administration of vitamin D3+ATRA may exert beneficial effects against diabetes-related cardiovascular consequences as a promising therapeutic agent by increasing miR-126 expression. However, no profitable effect was observed when vitamin D3 or ATRA was administered separately.

## Conflicts of interest

The authors have declared that there is no conflict of interest.
